# Change in Surface Conductivity of Elastically Deformed p-Si Crystals Irradiated by X-Rays

**DOI:** 10.1186/s11671-017-2210-x

**Published:** 2017-07-05

**Authors:** R. Lys, B. Pavlyk, R. Didyk, J. Shykorjak

**Affiliations:** Department of Sensor and Semiconductor Electronics, Ivan Franko National University of Lviv, 107, Tarnavskoho Str, Lviv, 79017 Ukraine

**Keywords:** Uniaxial deformation, X-ray irradiation, Dislocation, Defects restructuring

## Abstract

Changes in conductivity of irradiated and non-irradiated p-Si mono-crystals under the influence of elastic uniaxial mechanical stress were investigated in this paper. An analytical expression was suggested to describe the dependence of surface conductivity as a function of mechanical stress and X-ray irradiation dose. It was shown that 4-angular nano-particles on the surface of “solar” silicon affect the electroconductivity changes under mechanical stress. It was established that X-ray irradiation causes the generation of point defects in silicon. These defects suppress the dislocations movement. It was shown that the resistivity of previously irradiated samples of “electronic” silicon is only slightly sensitive to the influence of uniaxial compression at certain deformation rate.

## Background

A widespread use of semiconductor devices in the field of modern electronic technologies requires investigation of new semiconducting materials which possess high stability under the external influence such as X-ray irradiation and mechanical deformation. Nowadays, silicon is actively used in highly sensitive detectors and other semiconductor sensors operating in the radiation fields [1].

Most articles are dedicated to the impact of plastic deformation on the conductivity of n-Si [2, 3]. That is why the impact of elastic deformation on the properties of p-Si crystals is still considered to be an important scientific task. Redistribution of carrier concentration and impurities in deformed crystals are often characterized by the presence of dislocations which are effective getters of defects, especially on the surface of the crystal [4, 5]. It is known [3, 6] that the excitation of crystal electronic subsystems is also accompanied by the corresponding changes in the dislocation mobility. Excitation of the electronic subsystems could be as a result of external influence, such as radiation and electrostatic field. A characteristic feature of dislocations in silicon crystals is the presence of point defects (Cottrell cloud) with a high concentration around dislocations.

The surface of the crystals is the most sensitive to ionizing radiation. That is why the investigation of radiation-induced processes on the surface layers of silicon crystals is still considered to be relevant. The surface with the deposited Al-contacts is an effective getter for the structural defects [5–7]. Underneath the deposited metal film, the mechanical stresses appear due to inconsistencies in lattice parameters of the film and the semiconductor [5, 7]. These stresses stimulate the processes of defects gettering (impurity atoms, interstitial silicon atoms, and vacancies) in contact layer.

## Methods

**Fig. 1:**
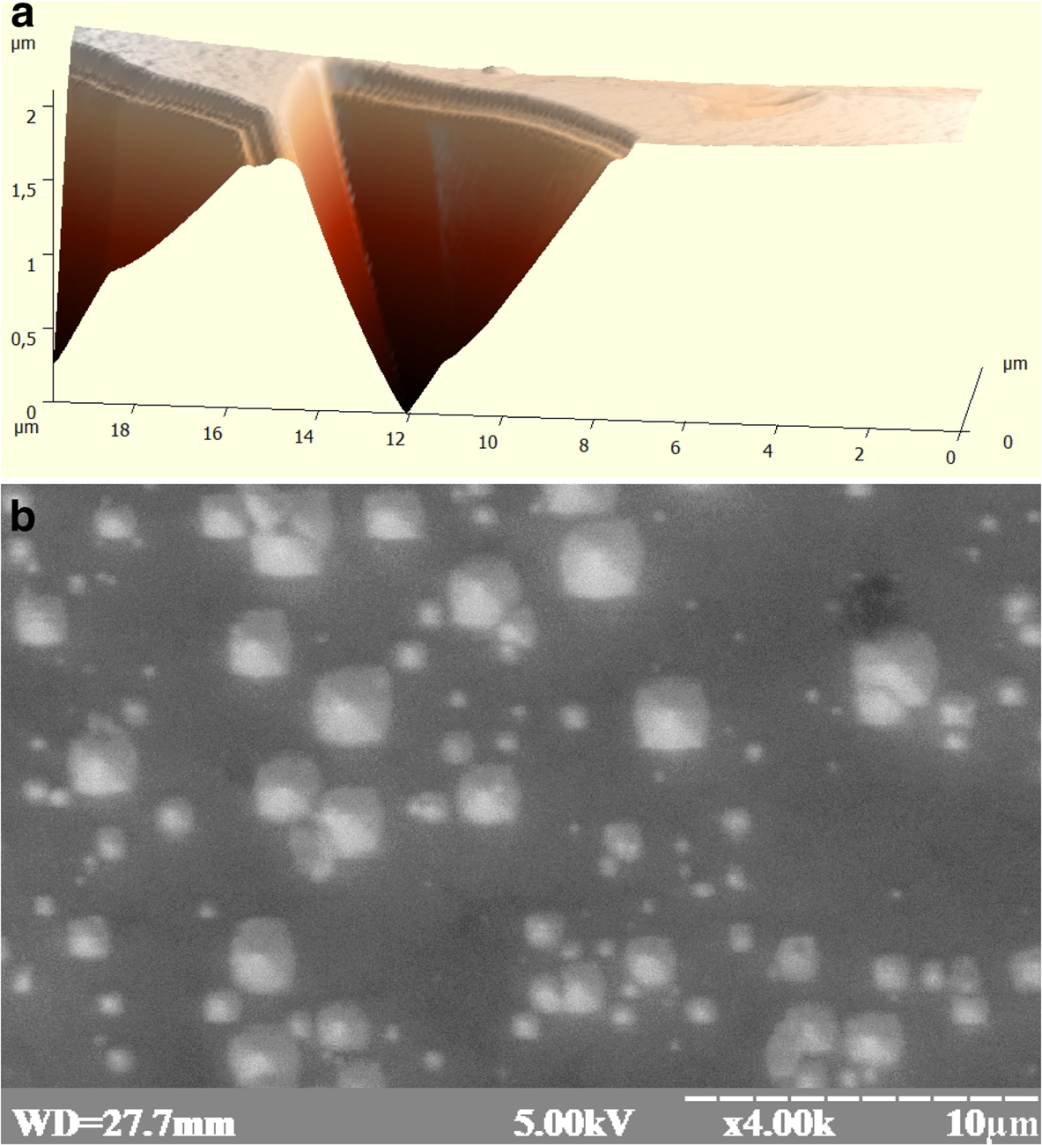
The appearance of the surface of the experimental samples: **a** appearance of dislocation etch pits on the surface of the p-Si crystal obtained in the field of atomic force microscope and **b** appearance of submicroscopic surface (111) of solar crystals

**Fig. 2:**
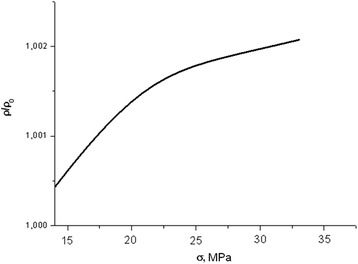
Dependence of longitudinal resistance of primary dislocation-free sample during the elastic deformation with the deformation rate 8 μm/min

Silicon mono-crystals of p-type conductivity, grown by Czochralski method (*ρ* = 10–20 Ω cm), were used in the research paper. These mono-crystals are of two types: (1) silicon for electronics—the so-called dislocation-free (or electronic) mono-crystals on the surface (111) of which, the concentration of triangular etch pits does not exceed 10^2^ cm^−2^ (Figs. 1a and 2), and (2) “solar” mono-crystals of silicon on the surface (111) of which, the defects in the form of 4-angular pyramids (Fig. 1b) were discovered due to a relatively large concentration of the background carbon (≈5 × 10^16^ cm^−3^) and oxygen (≈1.8 × 10^18^ cm^−3^) impurities. Four-angular pyramids are located in the same way. The size of the pyramid base is from 10 nm to 10 μm.

It was shown [8, 9] that in the formation of clusters, to which correspond 4-angular pyramidal etch holes can participate oxide layers of silicon, point defects, and layers with different structural states of silicon, particularly alpha silicon.

Experimental samples obtained dimensions 4 × 3.7 × 7.6 mm after sanding and chemical polishing. Ohmic contacts in the form of two strips with the width of 1.5 mm at the ends of the sample surfaces (111) were created by thermal evaporation of aluminum in a vacuum (10^−4^ Pa) at heated to 593 K sample. Measurement of electrical conductivity was conducted in a vacuum cryostat at residual gas pressure 10^−3^ Pa in the application of uniaxial compression to the ends (toward [$11\overline{2}$]) with a power of 15 to 40 MPa and a deformation rate of 8 or 32 μm/min. The samples were irradiated with a full range of X-radiation (*W*-anode, 50 kV, 10 mA), on both sides, on which aluminum contacts were coated. The distance between the source of X-rays and crystals was minimal (1–2 mm). It was found that the absorbed dose was increasing by 130 Gy in every 30 min. In the work, we firstly irradiated the experimental samples and, afterwards, we measured the resistance in the process of deformation.

## Results and Discussion

The research result on the change of induced mechanically conductivity along the direction of deformation (*ρ*(*σ*)) of “dislocation-free” samples of p-type conductivity under the influence of uniaxial stress (*σ*) is shown in Fig. 2. The increase of the load from 0 to 40 MPa (at the deformation rate 8 μm/min) lasts 45 min.

**Fig. 3:**
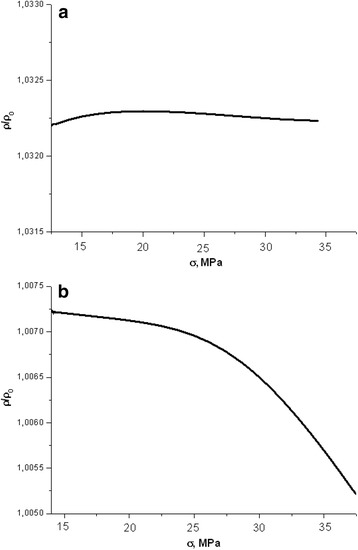
Dependence of longitudinal resistance of irradiated (*D* = 130 Gy) dislocation-free sample of silicon during elastic deformation with a growing strength of compression at a speed of 8 μm/min (**a**) and 32 μm/min (**b**)

In the process of deformation, the resistance of dislocation-free samples slightly increases. It should be noted that in the case of non-irradiated crystals, the change of deformation rate practically had no effect on the general view of dependences *ρ*(*σ*) [10, 11]. Similar dependences were obtained for irradiated samples (Fig. 3). An increase in resistance was observed after the action of X-irradiation. However, the nature of dependence *ρ*(*σ*) was observed to be somewhat different than for non-irradiated samples.

It can be seen that the resistance remains virtually unchangeable (Fig. 3a) during compression at a speed of 8 μm/min due to the effect of X-irradiation. The graphs of dependences of samples exposed to 260 and 480 Gy had a similar appearance. It was shown in previous studies [11] that the resistance was increasing proportionally to the square root of the absorbed dose during the irradiation process.

A fourfold increase in compression rate (from 8 to 32 μm/min) leads to the changes in the nature of the dependence of the resistivity on the load (Fig. 3b). There is a small (<0.2%) decrease in the resistance of irradiated samples in the process of compression. It ought to be noted that all measurements on conductivity changes were carried out with a high degree of accuracy (±0.045%) so that it was possible to correctly analyze small changes in resistivity in the experiment.

It should be noted that the dependence, shown in Fig. 3, has been received 7 days after the measuring of changes in the longitudinal resistance (*D* = 130 Gy) of dislocation-free samples at a speed of 8 μm/min (Fig. 3a). During the given time frame, the resistance almost got back to its original value, i.e., the value of resistance, which was observed after the irradiation and the application of mechanical stress.

**Fig. 4:**
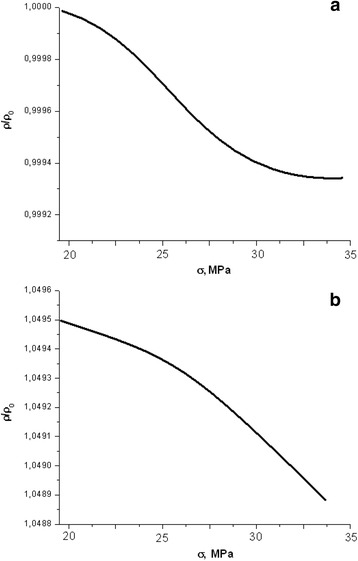
Dependence of longitudinal resistance of solar silicon during elastic deformation with a growing strength of compression: **a** *D* = 0 Gy, compression speed 32 μm/min; **b** *D* = 130 Gy compression speed 8 μm/min

Similar studies on measuring the resistance dependence on the action of elastic compression and after exposure to radiation were also conducted (Fig. 4) for experimental samples based on “solar silicon” of p-type conductivity, to which are inherent 4-angular pyramids on the surface (111).

First of all, it was found that the nature of resistance change of “solar” silicon of p-Si type on the size of the mechanical stress is independent of the speed of compression. A similar feature was observed in both non-irradiated and irradiated samples. The dependencies of the longitudinal resistance on mechanical stress changes by a relatively small value (<0.5%), and it slightly decreases under the increase in load (Fig. 4a).

Irradiation of experimental samples with X-rays (480 Gy) does not virtually affect the general nature of the change in longitudinal resistance of “solar” silicon during the elastic deformation (Fig. 4b). As for electronic samples, the resistance is proportional to the square root of absorbed dose [11]. During the mechanical stress, the resistivity decreases by a very small value (±0.1%).

**Fig. 5:**
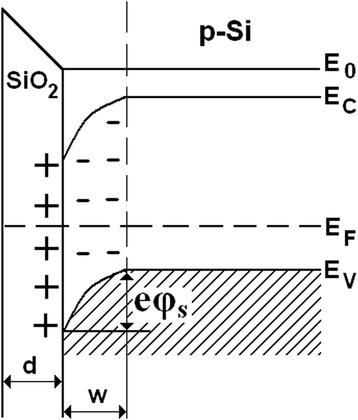
Distortion of the energy bands in the p-type conductor under provided positive charge at the interface of semiconductor-insulator

As it was shown in our previous studies [12, 13], the dielectric film SiO_2_ has a positive charge. Therefore, the space charge surface layer depleted in holes (with high resistance) and with a thickness *w* (Fig. 5) is created in silicon. The closer the Si-SiO_2_ to the interface, the fewer holes there are.

**Fig. 6:**
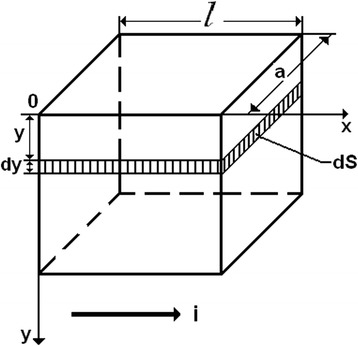
Calculations of conductivity

The concentration of holes in the surface layer of silicon and consequently its conductivity changes in case of the surface potential change (*φ*_*S*_). Let us consider a planar square plate (Fig. 6). Let the current flows parallel to the plane of the plate in the direction of one of its edges.

It was taken a thin parallel layer with thickness *dy* and cross-sectional area *dS* [14] at some distance (*y*) from the surface. This layer can be considered as homogeneous semiconductor, the resistance of which can be determined according to the following formula:

1 $$ d R=\rho \frac{l}{dS}, $$

2 $$ d S= a d y. $$

Since the plate is square (*l* = *a*), the conductivity of the layer is

3 $$ d\lambda =\frac{1}{dR}=\sigma d y, $$

where $\sigma =\frac{1}{\rho}$ is the electrical conductivity of the layer with *dy* thickness at *y* distance from the surface. For a p-type semiconductor, the conductivity can be written as $\sigma \approx e p(y){\mu}_p$. Then we get

4 $$ d\lambda = e p(y){\mu}_p dy. $$

Let us find the total surface conductivity (*λ*). You need to integrate the last expression in the range from zero to a thickness of several sustainable Debye screening, or, for example, restrict the width of the space charge region *w*:

5 $$ \lambda ={\displaystyle \underset{0}{\overset{w}{\int }} ep(y){\mu}_p dy= e{\mu}_p{\displaystyle \underset{0}{\overset{w}{\int }} p(y) dy}}. $$

In general, the concentration of holes in the depleted surface layer depends not only on the coordinates (*y*) but also on the applied mechanical stress (*σ*_*meh*_). It is determined by two components: *p*(*y*, *σ*_*meh*_) = *p*_1_(*y*) − *p*_2_(*σ*_*meh*_), where *p*_*1*_(*y*) is a component that corresponds to a change in carrier concentration with the change of distance from the surface of the semiconductor and *p*_*2*_(*σ*_*meh*_) is a component that shows by how much the concentration of holes is reduced due to their capture at dislocations during the mechanical stress. In addition, the mobility of holes is not a constant value. It depends on the mechanical stress. Therefore, the expression for the total surface conductivity can be written in the following form:

6 $$ \lambda = e{\mu}_p\left({\sigma}_{meh}\right){\displaystyle \underset{0}{\overset{w}{\int }}\left({p}_1(y)-{p}_2\left({\sigma}_{meh}\right)\right) dy}. $$

Mechanically induced change in conductivity can be written as follows:

7 $$ \lambda \left({\sigma}_{meh}\right)= e{\mu}_p\left({\sigma}_{meh}\right)\cdot \Big({\displaystyle \underset{0}{\overset{w}{\int }}{p}_1(y) dy-{\displaystyle \underset{0}{\overset{w}{\int }}{p}_2\left({\sigma}_{meh}\right) dy}\Big)= e{\mu}_p\left({\sigma}_{meh}\right)\cdot \left\{{\beta}_1-{\beta}_2\left({\sigma}_{meh}\right)\right\}}, $$

where $$ {\beta}_1={\displaystyle \underset{0}{\overset{w}{\int }}{p}_1(y) dy;\kern1em {\beta}_2\left({\sigma}_{meh}\right)={\displaystyle \underset{0}{\overset{w}{\int }}{p}_2\left({\sigma}_{meh}\right) dy}}={p}_2\left({\sigma}_{meh}\right){\displaystyle \underset{0}{\overset{w}{\int }} dy=} w\cdot {p}_2\left({\sigma}_{meh}\right) $$.

It should be noted that since *p*_*1*_(*y*) and *p*_*2*_(*σ*_*meh*_) also depend on irradiation effect, the factors *β*_*1*_, *β*_*2*_, and *μ*_*p*_ depend on the dose of X-irradiation. Therefore, the formula for surface conductivity before (*λ*(*σ*_*meh*_ *,0*)) and after (*λ*(*σ*_*meh*_,*D*)) irradiation can be written as follows:

8 $$ \lambda \left({\sigma}_{meh},0\right)= e{\mu}_p\left({\sigma}_{meh},0\right)\cdot \Big({\displaystyle \underset{0}{\overset{w}{\int }}{p}_1\left( y,0\right) dy- w\cdot {p}_2\left({\sigma}_{meh},0\right)\Big)= e{\mu}_p\left({\sigma}_{meh},0\right)\cdot \left\{{\beta}_1(0)-{\beta}_2\left({\sigma}_{meh},0\right)\right\}}. $$

9 $$ \lambda \left({\sigma}_{meh}, D\right)= e{\mu}_p\left({\sigma}_{meh}, D\right)\cdot \Big({\displaystyle \underset{0}{\overset{w}{\int }}{p}_1\left( y, D\right) dy- w\cdot {p}_2\left({\sigma}_{meh}, D\right)\Big)= e{\mu}_p\left({\sigma}_{meh}, D\right)\cdot \left\{{\beta}_1(D)-{\beta}_2\left({\sigma}_{meh}, D\right)\right\}}. $$

If the sample has a rectangular shape with a length (*l*) and width (*a*), we can write the final formula for the total surface conductivity as follows:

10 $$ \lambda \left({\sigma}_{meh}, D\right)=\frac{a}{l} e{\mu}_p\left({\sigma}_{meh}, D\right)\left\{{\beta}_1(D)-{\beta}_2\left({\sigma}_{meh}, D\right)\right\}, $$

where

11 $$ {\beta}_1(D)={\displaystyle \underset{0}{\overset{w}{\int }}{p}_1\left( y, D\right) dy,\kern1em }{\beta}_2\left({\sigma}_{meh}, D\right)= w\cdot {p}_2\left({\sigma}_{meh}, D\right). $$

The change of surface conductivity of irradiated p-Si crystals under the influence of mechanical stress is mainly determined by the change of three parameters: *β*_*1*_, *β*_*2*_, and *μ*_*p*_.

According to our previous studies, [10–13], the effect of X-ray exposure to the electronic silicon is accompanied by a slight increase in positive charge in the dielectric surface layer of SiO_2_. As a result, the factor *β*_*1*_: *β*_*1*_(*D*) > *β*_*1*_(*0*) slightly increases. For the “solar” silicon, opposite dependences are observed: *β*_*1*_(*D*) < *β*_*1*_(*0*).

Regarding the factor *β*_*2*_, its changes are mainly determined by the change of *p*_*2*_(*σ*_*meh*_ *, D*) under radiation effect. These changes are much more substantial compared to the change of the parameter *β*_*1*_. X-irradiation triggers the generation of point defects in silicon, which act as stoppers for dislocation motion. As a result, after the exposure to radiation, the factor *β*_*2*_ for these samples sharply decreases (reduces the number of dislocation trapped holes) for both types of experiment samples p-Si: *β*_*2*_(*D*) < *β*_*2*_(*0*).

In non-irradiated crystals of “solar” silicon, the existent defects, to which correspond 4-angular pyramidal etch pits, play the role of stoppers for dislocation motion. Additional defects generated by irradiation did not play a significant role in the background of a strong concentration of existent surface defects.

The mobility of holes slightly reduces during exposure to radiation due to the increase in the scattering at radiation defects: *μ*_*p*_(*D*) < *μ*_*p*_(*0*). By this mechanism can be explained the experimentally confirmed decrease in the conductivity of irradiated samples of silicon. Thus, an analysis of the formulas (8) and (9) confirms the growth of the resistance under the increase of the value of the absorbed dose of X-irradiation mainly by reducing the mobility (*μ*_*p*_(*D*) < *μ*_*p*_(*0*)) and the concentration of free charge carriers—holes (*β*_*2*_(*D*) < *β*_*2*_(*0*)).

If we consider the equation data at a fixed dose, we can draw following conclusions, which confirm the above given experimental dependences of the resistance to mechanical stress:

1. The resistance of non-irradiated samples of electronic silicon increases under compression (Fig. 2). This occurs due to significant growth of factor *β*_*2*_ under the action of mechanical stress. During compression, the change (growth) of factor *β*_*2*_ significantly exceeds the change (increase) in mobility of holes under the increase of mechanical stress. As to the parameter *β*_*1*_, its value does not depend on *σ*_*meh*_.
  So, in our case, the increase of resistance (compression) and decrease (decompression) of the load for non-irradiated crystals based on electronic silicon can be explained by the movement of dislocations, which are taking over the major carriers. Forces that cause movement of defects, the coagulation of clusters, and the condensation of clusters on dislocations [15] appear in the elastically deformed crystal lattice. Defects are becoming centers of capturing major carriers while coagulating into larger clusters as micro-pores, clusters of internode silicon, and impurities. The accumulation of defects in the surface layer of silicon tends to reduce its conductivity. It is displayed by corresponding growth factor *β*_*2*_ in the formula for the surface conductivity.
2. The resistance of irradiated samples of electronic silicon slightly changes under compression (Fig. 3a). This is caused by the reduction of change (growth) of factor *β*_*2*_, due to impeded dislocation motion. In other words, mutually competing changes of parameters *β*_*2*_ and *μ*_*p*_ are commensurate under compression of irradiated samples of electronic silicon.
  Resistance reduction of electronic silicon crystals in combination with stress increase (Fig. 3b) occurs due to the decrease in the longitudinal effective mass of heavy holes [16, 17] and a corresponding increase in their mobility under compression. This is displayed by corresponding mobility increase *μ*_*p*_ in the formula for the surface conductivity.
3. The resistance of irradiated and non-irradiated samples of solar silicon slightly decreases under compression (Fig. 4). Dislocation motion process is very difficult for these experimental samples. Moreover, additional defects that are moving from the middle to surface of silicon make the unessential contribution to the current transport on the background of high concentration of existing surface defects in crystals of solar silicon. The action of radiation additionally increases the concentration of surface defects in crystals of solar silicon. Thus, mutually competing changes of parameters *β*_*2*_ and *μ*_*p*_ are commensurate under compression of irradiated and non-irradiated samples of solar silicon.
  Effect of radiation (Fig. 4b) additionally increases the concentration of surface defects in crystals of solar silicon. Therefore, additional defects, which are moving due to gettering, make a small contribution to the current transport than it for not-irradiated crystals.

## Conclusions

Two main factors that affect the resistance of p-Si crystal have to be considered during the mechanical load. The first factor is an increase in resistance with an increase of load (compression) and a decrease of resistance with a decrease of load (unclasping) due to the process of dislocations movement, which is taking over the major carriers. The second factor is the decrease of resistance of silicon crystals with an increase in load due to the decrease in effective mass of holes and a corresponding increase in their mobility.

X-ray irradiation causes the generation of vacancies and interstitial atoms in silicon, which act as stoppers for the movement of dislocations. Due to the increase in scattering of radiation defects, the mobility of holes slightly decreases during X-ray irradiation. In non-irradiated crystals of solar silicon, the existent defects play the role of stoppers for the movement of dislocations.

Pre-irradiated experimental p-Si crystals ( electronic and “solar-based” silicon) have a property to slightly change its resistivity (±0.2%) under the influence of uniaxial compression (speed of stress supply 8 μ/min), within the elastic deformation along the stream [$11\overline{2}$].

In the pre-irradiated electronic p-Si samples, the dependence of the resistance on uniaxial mechanical stress significantly depends on the rate of compression. At a low velocity of stress supply (8 μm/min), the resistance increases with the increase of mechanical stress; at high speeds (32 μm/min), decreases. For crystals based on solar p-type silicon, the dependence of resistance under mechanical loading is independent of the compression rate.

## Abbreviations

*ρ*(*σ*): The change of induced mechanically conductivity along the direction of deformation
